# *Enterocytozoon bieneusi* Identification Using Real-Time Polymerase Chain Reaction and Restriction Fragment Length Polymorphism in HIV-Infected Humans from Kinshasa Province of the Democratic Republic of Congo

**DOI:** 10.1155/2012/278028

**Published:** 2012-07-01

**Authors:** Roger Wumba, Menotti Jean, Longo-Mbenza Benjamin, Mandina Madone, Kintoki Fabien, Zanga Josué, Sala Jean, Kendjo Eric, Guillo-Olczyk AC, Thellier Marc

**Affiliations:** ^1^Département de Médecine Tropicale, Maladies Infectieuses et Parasitaires, Cliniques Universitaires de Kinshasa, Université de Kinshasa, 747 Kinshasa XI, Democratic Republic of Congo; ^2^Service de Parasitologie-Mycologie, Hôpital Saint-Louis, Assistance Publique-Hôpitaux de Paris et Faculté de Médecine Lariboisière-Saint-Louis, Université Paris VII, 75010 Paris, France; ^3^Faculty of Health Sciences, Walter Sisulu University, Private Bag XI, Mthatha, Eastern Cape 5117, South Africa; ^4^Department of Internal Medicine, University of Kinshasa, 783 Kinshasa XI, Democratic Republic of Congo; ^5^National Center for Malaria Research, AP-HP, CHU Pitié Salpetrière, 75013 Paris, France; ^6^AP-HP, Groupe hospitalier Pitié-Salpêtrière, Service de Parasitologie-Mycologie, Université Pierre et Marie Curie, 75013 Paris, France

## Abstract

*Objective*. To determine the prevalence and the genotypes of *Enterocytozoon bieneusi* in stool specimens from HIV patients. *Methods*. This cross-sectional study was carried out in Kinshasa hospitals between 2009 and 2012. Detection of microsporidia including *E. bieneusi* and *E. intestinalis* was performed in 242 HIV-infected patients. Typing was based on DNA polymorphism of the ribosomal DNA ITS region of *E. bieneusi*. PCRRFLP generated with two restriction enzymes (Nla III and Fnu 4HI) in PCR-amplified ITS products for classifying strains into different lineages. The diagnosis performance of the indirect immune-fluorescence-monoclonal antibody (IFI-AcM) was defined in comparison with real-time PCR as the gold standard. *Results*. Out of 242 HIV-infected patients, using the real-time PCR, the prevalence of *E. bieneusi* was 7.9% (*n* = 19) among the 19 *E. bieneusi*, one was coinfected with *E. intestinalis*. In 19 *E. bieneusi* persons using PCR-RFLP method, 5 type I strains of *E. bieneusi* (26.3%) and 5 type IV strains of *E. bieneusi* (26.3%) were identified. The sensitivity of IFI-AcM was poor as estimated 42.1%. *Conclusion*. Despite different PCR methods, there is possible association between HIVinfection, geographic location (France, Cameroun, Democratic Republic of Congo), and the concurrence of type I and type IV strains.

## 1. Introduction

It is established that *Enterocytozoon bieneusi* (*E. bieneusi*) is the most commonly characterized microsporidia species among human beings. Microsporidia, obligate intracellular parasites, lack eukaryotic ribosomal features and peroxisomes [1]. Their spores do penetrate and infect eukaryotic cells in various invertebrate and vertebrate organisms. The literature reports epidemiology, causes, diagnosis, and digestive disorders related to microsporidiosis among HIVpatients [2–7].

In Kinshasa region, The capital city of The Democratic Republic of Congo (DRC), we detected *E. bieneusi* infection in HIV patients using only light microscopy and Fungi Fluor [8] as well as conventional polymerase chain reaction (PCR) method [9]. We could confirm the sensitivity of the diagnosis of *E. bieneusi* infection by a real-time PCR assay in comparison with traditional methods [10, 11].

*E. bieneusi *genotypes were also identified by PCR-restriction fragment length polymorphism (RFLP) analysis [12, 13].

Therefore, the objective of this study was to determine the prevalence and the genotypes of *E. bieneusi* in stool specimens among HIV patients by developing a rapid and efficient real-time PCR and PCR-RFLP approach.

## 2. Materials and Methods

### 2.1. Study Design

This study was designed as a descriptive cross-sectional approach between December 2009 and January 2012.

### 2.2. Ethical Considerations

The institutional review boards and the Committee of Ethics of the University of Kinshasa Faculty of Medicine approved the protocol of the study which was conducted in compliance with the principles of Helsinki Declaration. The procedures of the study were explained, and an informed consent sheet was signed by each participant or a designated literate substitute when necessary.

### 2.3. Study Setting

In the Kinshasa community, Democratic Republic of Congo, the Cliniques Universitaires de Kinshasa (CUK) as the teaching hospital at the south-western part of Kinshasa city, the general referral hospital of Kinshasa (HGRK) in the center of Kinshasa city, the general referral hospital of Kintambo (HGRKint) at the Northeastern Kinshasa city, and military referral hospital of Camp Kokolo (HMRK) at the western part of Kinshasa city were randomly selected.

### 2.4. Patients and Clinical Specimens

We included 242 consecutive HIV-infected patients. The clinical signs characteristic of HIV disease were collected among all participants.

### 2.5. Diagnosis of *E. bieneusi* Infection

We collected 242 fresh stool samples in pH 7.2 buffer stored at + 4°C before analysis. The stool specimens from all 242 patients were diluted at PBS solution for microscopic examination. 

Microscopic examination and specific staining were done both in Kinshasa University Parasitology laboratories (CUK) and in the Pitié Salpêtrière Hospital (PSL) Parasitology Mycology Laboratory, Paris, France. Stool samples (one for each patient) were studied using optical microscopy (direct examination and trichrome specific staining as modified by Weber) for microsporidia detection [14].

The indirect immunofluorescence-monoclonal antibody (IFI-AcM) techniques were used for the identification of *E. bieneusi* and *E. intestinalis* [15, 16].

### 2.6. Genomic DNA Extraction

DNA extraction was performed by using the QIAamp DNA Mini Kit (Qiagen, Hilden, Germany) according to the supplier's protocol.

### 2.7. Real-Time PCR

We carried out a real-time PCR for all samples at the Saint Louis Hospital Parasitology Mycology service in Paris, France, using a 7500 Real-Time PCR System (Applied Biosystems, Foster City, CA, USA) for all three species identification (*E. bieneusi * and* E. intestinalis*). 

For *E. bieneusi*, the real-time PCR assay amplified a 102bp fragment of the small subunit ribosomal RNA gene, with FEB1 (5′-CGCTGTAGTTCCTGCAGTAAACTATGCC-3′) and REB1 (5′-CTTGCGAGCGTACTATCCCCAGAG-3′) primers and a fluorescent TaqMan probe (5′-ACGTGGGCGGGAGAAATCTTAGTGTTCGGG-3′), as previously described [10]. For *E. intestinalis*, the real-time PCR assay was performed by using FEI1 (5^'^-GCAAGGGAGGAATGGAACAGAACAG-3′) and REI1 (5′-CACGTTCAGAAGCCCATTACACAGC-3′)-primers, with the following fluorescent TaqMan probe: 5′-FAM-CGGGCGGCACGCGCACTACGATA-TAMRA-3′, as previously described [10, 11].

### 2.8. PCR-RFLP for *E. bieneusi* Genotype Identification

The PCR-RFLP assay was performed on a 9700 PCR system (Applied Biosystems) as previously described [12, 13]. The RFLP analysis was performed on a 2% agarose gel by comparing the number and the length of the obtained PCR undigested and digested fragments by using Fnu4HI and NlaIII restriction enzymes.

### 2.9. Statistical Analysis

Data were expressed as proportions (%) for categorical variables and means with standard deviations for continuous variables. Differences were compared by the chi-square test for proportions and by the Student's *t*-test for continuous variables with results considered statistically significant for *P* value <0.05. All analyses were performed by use of STATA (version 11) software package. 

## 3. Results 

### 3.1. Clinical Profile of Patients

Of 242 HIV/AIDS patients, 35.9% (*n* = 87) were males and 64.1% (*n* = 155) were females: sex ratio of 2 women: 1 man. The mean age of the participants was 39.2 ± 11.8 years (range: 15–73).

Table 1 presents the clinical signs of the study population. Asthenia and diarrhea were the most frequent signs among the participants. 

### 3.2. Molecular Evaluation and Prevalence

Out of 242 HIV-infected patients, using the real-time PCR, the prevalence of *E. bieneusi* was 7.9% (*n* = 19). Among the 19 *E. bieneusi*, one was coinfected with *E. intestinalis*.

Table 2 presents the findings from IFI-AcM, real-time PCR, and genotypes. The diagnosis efficiency of IFI-AcM was defined with comparison with the real-time PCR as follows: sensitivity of 42.1%, specificity of 100%, positive predictive value of 100%, and negative predictive value of 95%.

**Figure 1:**
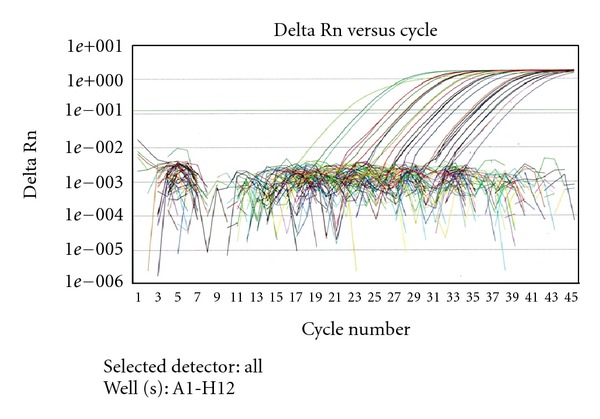
Amplification curves obtained with the *E. bieneusi*-specific real-time PCR assay.

Figure 1 shows the function of the relative fluorescent signal (Delta Rn) according to the cycle number.

**Figure 2:**
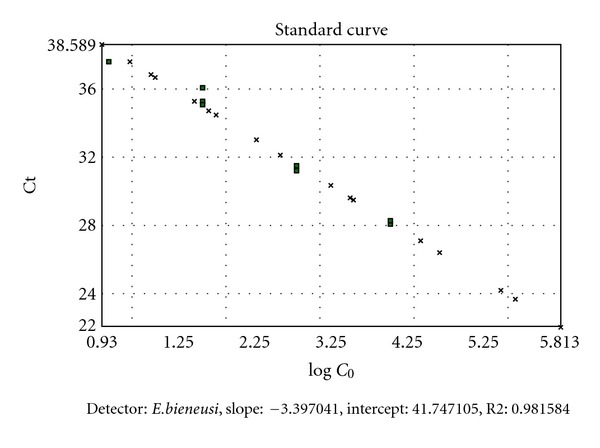
Standard curve representing the threshold cycle (Ct) values as a function of the decimal logarithms of *E. bieneusi* small subunit rRNA gene copy number per *μ*l.

The sensitivity and reproducibility of real-time PCR was assessed by repeated testing of serial dilutions (Figure 2). The relation between Ct value and the decimal logarithm of *E. bieneusi* small subunit rRNA gene copy number per *μ*l was as follows: slope = −3.397 and intercept = 41.747. 

**Figure 3:**
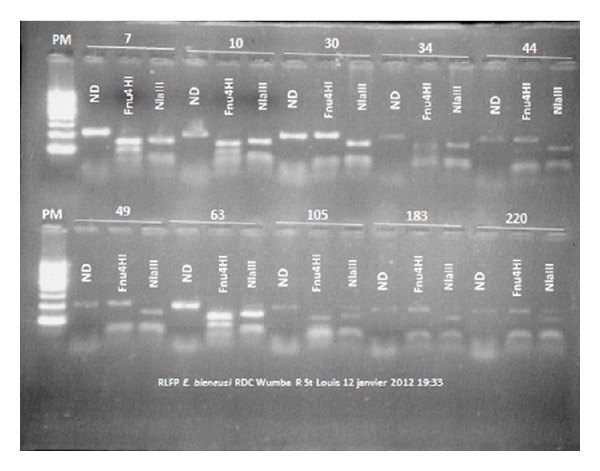
RFLP analysis of *E. bieneusi* PCR products after digestion with Fnu4HI and NlaIII enzymes. ND: not digested, PM: molecular weight marker.

PCR-RFLP analysis of the amplification products of the ITS region was then performed on the 19 *E. bieneusi* stool isolates (Figure 3). We found two genetically unrelated lineages: type I strains without digestion of amplicons with Fnu 4HI, and type IV strains with digestion of amplicons with NlaIII and Fnu4HI.

## 4. Discussion

In the present study, we have used two real-time PCR assays and a PCR-RFLP assay for the quantitative detection of *E. bieneusi* DNA and strain genotyping from stool specimens. 

Clinical features from the HIV-infected participants were similar to the frequency of diarrhea reported among other African patients [2–7].

The prevalence of *E. bieneusi* identified by PCR in these HIV Congolese patients was estimated at 8.2% (7.9% of *E. bieneusi*), which was higher than the prevalence of microsporidia found using similar PCR techniques in other African countries (less than 5%) [4, 17–25]. These low rates of microsporidiosis could be related to the location and availability of antiretroviral therapy (ART). Indeed, the prevalence of microsporidia including *E. bieneusi* in HIV-infected people has dramatically decreased in countries where ART is widely available [26, 27]. However, in most African countries including our Congolese study, few patients have access to ART [1, 8, 9], which could explain the higher prevalence found in our study and in some other African studies among HIV-infected individuals [8, 9, 28].

In this study, we used a rapid and efficient qPCR method combined with PCR-RFLP genotyping and IIF-MAb for determining intestinal microsporidiosis from stool specimens among HIV-infected patients. Thus, we confirmed the best diagnostic of *E. bieneusi* using more sensitive and specific real-time PCR than the diagnosis of *E. intestinalis* [10–13].

The literature reports that *E. bieneusi* is a relatively homogeneous entity with PCR-RFLP-based putative polymorphism of the ITS region of *E. bieneusi* [5]. This putative polymorphism of the ITS region of *E. bieneusi* had a genetic diversity of *E. bieneusi* [5].

Among the 19 *E. bieneusi* cases we studied, we identified 5 type I strains of *E. bieneusi* (26.3%) and 5 type IV strains. By contrast, HIV-infected patients in France were in majority infected with type I strains [12, 13]. Interestingly, type IV strains were also encountered in a previous study in Cameroon [18]. Furthermore, Tumwine et al. found a majority of genotype K strains, which correspond to type IV in our classification, in children from Uganda [29].

### 4.1. Findings and Current Understanding in the Field within the Field

The present work and the work by Liguory et al. [12, 13] were performed using the same PCR-RFLP developed by Liguory team. Our typing was based on DNA polymorphism of the ribosomal DNA internal transcribed spacer (ITS) region of *E. bieneusi*. PCR-RFLP generated with two restriction enzymes (Nla III and Fnu4HI) in PCR-amplified ITS products at classifying type I, type II, type III, and type IV [12, 13].

Santín et al. [30] were among the leaders to reduce confusion associated with the identification of genotypes within *E. bieneusi* after the meeting during IWOP-10. According to the consensus [30], previously, the correspondence for the nomenclature was as follows: genotype B belongs to type I, genotype C belongs to type II, genotype, undetermined genotype does not belong to type III, and genotype K belongs to genotype IV [13, 30, 31].

Despite the standard methods for determining the genotypes of *E. bieneusi* based on the DNA sequence of the internal transcribed spacer (ITS) region, the r-RNA gene in the publication of Santín et al. [30], the present work in Kinshasa (DRC), and the previous works in France [12, 13] and in Cameroun [31] showed a significant association between HIV-infection and genotypes I and IV *E. bieneusi*. Genotype IV *E. bieneusi* was only present among HIV-patients from Nigeria [32], Uganda [29], Gabon [31], and Portugal [33]. The genotypes II and III *E. bieneusi* were not identified in the present study from Kinshasa (DRC) as they are not yet reported from Africa. However, genotypes II and III *E. bieneusi* are more frequent among HIV-negative people from Europe [12, 13]. Genotype I in HIV-patients is commoner and more frequent than genotype IV in Europe [12, 13, 34] than in HIV-patients from Central Africa including Democratic Republic of Congo with the present study and Cameroun [31].

In this study, the genotype I–genotype IV *E. bieneusi* ratio was 1 in HIVpatients and emerging: genotype I *E. bieneusi* in 5 cases of HIV/AIDS versus genotype IV *E. bieneusi* in 5 cases of HIV/AIDS. Possible rapid travels between France and francophone Central Africa may be a factor contributory to the emerging genotype I *E. bieneusi*.

### 4.2. Implications for Public Health

The significant diagnosis efficiency of PCR methods for *E. bieneusi* will have implications on management of HIV-related microsporidia.

The accurate identification and differentiation of microsporidian species by real-time PCR techniques will improve therapy, clinical manifestations, and prognosis [35–37].

Modes of transmission and sources of human infection by *E. bieneusi* or HIV and molecular analyses developed by real-time PCR and RFLP should be useful for epidemiological studies [1, 5, 8, 9, 35–39].

## 5. Conclusion 

The prevalence of *E. bieneusi* is emerging. We used a sensitive, specific, rapid, and efficient approach for typing *E. bieneusi* obtained from stool specimens by real-time PCR and PCR-RFLP assays. Genotype I *E. bieneusi* is more prevalent among HIV-patients from Europe than the genotype I–genotype IV *E. bieneusi* estimated 1 in HIV-infected patients from the present study in Kinshasa, Democratic Republic of Congo.

## Figures and Tables

**Table 1:** Clinical signs of our HIV patients.

Clinical signs	*N*/242	%
Asthenia	88	36,3
Diarrhea	83	34,3
Pulmonary signs	52	21,4
Cutaneous signs	42	17,3
Anorexia	28	11,5
Fever	25	10,3
Emaciation	14	5,7
Anemia	5	2

**Table 2:** Microsporidia (*E. bieneusi*, *E. intestinalis,* and genotypes).

*N*/19	IFI-AcM *Eb,Ei *	PCR RT *Eb,Ei *	Genotypes par RFLP
07	*Eb*	*Eb*	**Type 4**
08	No	*Eb *	**ND**
10	*Eb *	*Eb*	**Type 4**
12	No	*Eb*	**ND**
30	No	*Eb*	**Type 1**
34	*Eb*	*Eb*	**Type 4**
36	*Eb*	*Eb*	**ND**
37	*Eb*	*Eb*	**ND**
39	*Eb*	*Eb*	**ND**
40	*Eb*	*Eb*	**ND**
44	No	*Eb*	**Type 1**
49	No	*Eb*	**Type 1**
**63**	***Eb***	***Eb, Ei***	**Type 4**
89	No	*Eb*	**ND**
93	No	*Eb*	**ND**
105	No	*Eb*	**Type 4**
134	No	*Eb*	**ND**
183	No	*Eb*	**Type 1**
220	No	*Eb*	**Type 1**
